# Bronchial hyperresponsiveness testing in athletes of the Swiss Paralympic team

**DOI:** 10.1186/2052-1847-5-7

**Published:** 2013-04-15

**Authors:** Mirjam Osthoff, Franz Michel, Matthias Strupler, David Miedinger, Anne B Taegtmeyer, Jörg D Leuppi, Claudio Perret

**Affiliations:** 1 Clinic of Internal Medicine, University Hospital Basel, Petersgraben 4, Basel, CH- 4031, Switzerland; 2 Institute of Sports Medicine, Swiss Paraplegic Center, Nottwil, Switzerland; 3 Clinic of Pharmacology and Toxicology, University Hospital Basel, Basel, Switzerland; 4 Faculty of Medicine, University of Basel and University Clinic of Internal Medicine, Kantonsspital Baselland, Liestal, CH4410, Switzerland

**Keywords:** Disability, Eucapnic voluntary hyperventilation, Exercise-induced bronchoconstriction, Spinal cord injury, Mannitol challenge, Paraplegia

## Abstract

**Background:**

The aim of this study was to assess airway hyperresponsiveness to eucapnic voluntary hyperventilation and dry powder mannitol challenge in athletes aiming to participate at the Paralympic Games 2008 in Beijing, especially in athletes with spinal cord injury.

**Methods:**

Forty-four athletes with a disability (27 with paraplegia (group 1), 3 with tetraplegia (group 2) and 14 with other disabilities such as blindness or single limb amputations (group 3) performed spirometry, skin prick testing, measurement of exhaled nitric oxide, eucapnic voluntary hyperventilation challenge test (EVH) and mannitol challenge test (MCT). A fall in FEV1 of ≥10% in either challenge test was deemed positive for exercise-induced bronchoconstriction.

**Results:**

Fourteen (32%) athletes were atopic and 7 (16%) had a history of physician-diagnosed asthma. Absolute lung function values were significantly lower in patients of group 1 and 2 compared to group 3. Nine (20%) athletes were positive to EVH (8 paraplegics, 1 tetraplegic), and 8 (18%) athletes were positive to MCT (7 paraplegics, 1 tetraplegic). Fourteen (22.7%) subjects were positive to at least one challenge; only three athletes were positive to both tests. None of the athletes in group 3 had a positive test. Both challenge tests showed a significant association with physician-diagnosed asthma status (p = 0.0001). The positive and negative predictive value to diagnose physician-diagnosed asthma was 89% and 91% for EHV, and 75% and 86% for MCT, respectively.

**Conclusion:**

EVH and MCT can be used to identify, but especially exclude asthma in Paralympic athletes.

## Background

Athletes participating at the Paralympic Games 2008 in Beijing had to apply for a therapeutic use exemption if they wished to use inhaled beta agonists as these were prohibited substances according to the World Anti-Doping Code Prohibited List [1]. They had to provide objective criteria for the existence of exercise-induced bronchoconstriction or exercise induced asthma according to the World Antidoping Agency’s Therapeutic Use Exemption Guidelines [2].

As exercise challenge tests are quite insensitive to identify exercise induced bronchoconstriction in elite athletes [3], easier but also standardized tests would be needed. In addition, standardization with respect to both workload and environmental conditions is almost impossible to achieve for sport-specific exercises in the field [3], and even more so for disabled athletes. Provocation challenge tests such as EVH and the MCT have therefore been used to assess exercise-induced bronchoconstriction and asthma in athletes in a standardized fashion. EVH, where athletes are required to hyperventilate a dry air mixture at room temperature for six minutes, has been established as a standardized test to diagnose exercise-induced asthma [3,4] and has been recommended in elite athletes [5,6]. MCT tests athletes’ response to inhalation of dry powder mannitol from a handheld device in increasing doses and has also been shown to identify individuals with exercise-induced bronchoconstriction and asthma [7-9] and correlated well with EVH in able-bodied athletes [10].

Whether these tests can be used to assess athletes with a disability, however, is unknown. We therefore set out to test and compare EVH and MCT and their utility to diagnose exercise-induced asthma in elite athletes with a disability, in particular in athletes with spinal cord injury.

## Methods

Athletes with a disability from the Swiss national team who were planning to participate in the Paralympic Games 2008 in Beijing were asked to take part in the study. The athletes answered questions about asthma, underwent skin prick testing, spirometry, and exhaled nitric oxide (NO) measurement and were subsequently challenged with EVH and MCT on two separate occasions. The study was approved by the Ethics Committee of the Canton Lucerne, Switzerland. All participants gave written informed consent and the study was carried out in accordance with the Declaration of Helsinki [11].

### Subjects

Forty-four athletes with a disability were studied. Twenty-seven (61.4%) were paraplegic (Group 1), 3 (6.8%) tetraplegic (group 2), and 14 (31.8%) with other disabilities such as blindness or single limb amputation (group 3). All athletes were non-smokers. The diagnosis of asthma was based on previous physician-diagnosed asthma (a positive answer to the following two questions: “Have you ever had asthma?” and “Was this confirmed by a doctor?” and on current symptoms) [12].

### Skin-prick test

Each athlete underwent skin-prick testing to common aeroallergens according to the SAPALDIA protocol [13]. Atopy was defined as having a positive reaction to one of the allergens on skin-prick testing [13].

### Lung function testing

Baseline lung function was performed in each athlete in the sitting position using a nose clip according to standardized guidelines [14]. For the measurement a MasterScreen Body (Viasys Healthcare GmbH, Hoechberg, Germany) was used. The spirometer was calibrated according to the suggestions of the producer. The following parameters were measured (total lung capacity (TLC), residual volume (RV), functional residual capacity (FRC), vital capacity (VC), endexpiratory reserve volume (ERV), forced vital capacity (FVC), forced expiratory volume in one second (FEV1)).

### Exhaled nitric oxide

Orally exhaled nitric oxide (FeNO) measurements were performed prior to lung function and bronchial challenge testing according to published guidelines [15,16] using a device with a built-in biofeedback mechanism (NIOX MINO, Aerocrine AB, Solna, Sweden) at a flow rate of 50 mL/s.

### Bronchoprovocation challenge tests

**Figure 1:**
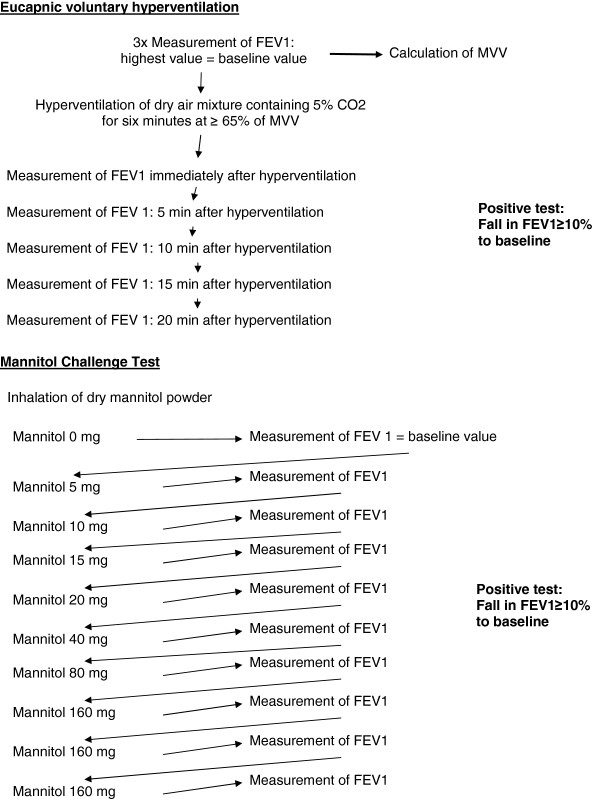
**Bronchoprovocation challenge tests.** MVV = maximal voluntary volume; FEV 1 = forced expiratory volume in one second.

Each athlete underwent an EVH challenge test and a MCT on separate days. Athletes with previous physician-diagnosed asthma were asked to abstain from their regular medication one week prior to lung function testing. Exercise-induced bronchoconstriction was defined as a fall in FEV1 of ≥10% in either EVH challenge test and/or MCT [9,17] (Figure 1).

1. Eucapnic voluntary hyperventilation

Before each test FEV1 was measured three times and the highest value was taken as the baseline value for determining maximal voluntary volume (MVV). MVV was calculated as 35 × FEV1. Athletes were then instructed to hyperventilate at minimum level of 65% of their MVV for six minutes breathing a gas mixture containing 5% CO_2_ and 21% O_2_. The inhaled gas mixture was kept constant using the Eucap Sys™ (SMTEC SA, Nyon, Switzerland). Immediately following the challenge, FEV1 was measured twice and the highest value was taken. Measurements were repeated after 5, 10, 15 and 20 minutes. A fall of 10% or greater to baseline in one time point was considered as positive [17].

2. Mannitol challenge

MCT was performed by administering dry mannitol powder (Aridol™ Pharmaxis Ltd, Sydney, Australia), inhaled from a dry powder device (Osmohaler, Pharmaxis Ltd, Frenchs Forest, NSW, Australia) as described previously by Anderson et al. [18]. FEV1 was measured 60 seconds after the delivery of each dose (5, 10, 20, 40, 80, 160, 160, 160 mg). The test continued until the FEV1 had fallen 15% or the maximum cumulative dose of 635 mg had been administered. A fall in FEV1 greater or equal to 10% was noted positive for mannitol-induced bronchoconstriction. PD_10_ (‘provoking dose-10’) was defined as the cumulative dose of mannitol causing a fall of 10% in FEV1. The response–dose ratio (RDR) for mannitol was calculated by dividing the highest percentage fall in FEV1 by the cumulative mannitol dose needed to cause this fall. RDR was compared to the fall in FEV1 in the EVH challenge.

### Statistical analysis

Differences in subjects’ characteristics were analysed using one way ANOVA, the Χ^2^ test or Fisher’s exact test where appropriate. Interrater reliability (Kappa) was used to examine the agreement between EVH and MCT. A p-value of 0.05 or less was defined as statistically significant. Geometric means were calculated using the log transformed values of PD_10_ and RDRs. Responses to EVH and MCT were compared using Spearman correlation coefficients. Statistical analyses were performed using GraphPad Prism Version 5.01 for Windows.

## Results

### Subjects

Of the forty-four participating athletes twenty-seven (61.4%) were paraplegic (group 1), 3 (6.8%) tetraplegic (group 2) and 14 (31.8%) had other disabilities such as blindness or single limb amputation (group 3). Fourteen (31.8%) athletes were found to be atopic and seven (15.9%) subjects had a history of physician diagnosed asthma; eleven athletes (25%) were diagnosed with asthma after assessment. The three groups did not show any statistically significant differences in the mean age, weight, height or gender distribution. Individual patient characteristics are given in Table 1.

**Table 1:** Subject characteristics

**Baseline characteristics**	**Group 1**	**Group 2**	**Group 3**	**All**	**p-value**
	**(Paraplegics)**	**(Tetraplegics)**	**(Single limb amputees; blind athletes)**		
Number of athletes	27	3	14	44	
Males (N, %)	16 (59.3%)	2 (66.7%)	12 (85.7%)	30 (68.2%)	0.23
Mean age years (SD)	34.1 (±12.2)	42.3 (±9.3)	33 (±8.6)	34.4 (±11)	0.45
Mean height cm (SD)	170 (±12.9)	166.3 (±15.6)	177.1 (±6.7)	172 (±11.8)	0.06
Mean weight kg (SD)	63.5 (±14.4)	63.7 (±25.1)	71 (±12.7)	65.9 (±14.7)	0.16
Atopy (N, %)	12 (44.4)	0 (0.0)	2 (14.3)	14 (31.8)	0.07

Legend: *SD* = standard deviation; p-values are given for the three neurologically defined groups.

### Baseline lung function

Results of baseline lung function testing are shown in Table 2. As there are no standardized predicted values for paraplegics and tetraplegics, we used absolute values for comparison. Statistically significant differences were found between the three defined groups for VC, ERV, FVC, and FEV1, with tetraplegics having the lowest values for vital capacity and FEV1.

**Table 2:** Baseline lung function

	**Group 1**	**Group 2**	**Group 3**	**All**	**p-value**
	**(Paraplegics)**	**(Tetraplegics)**	**(Single limb amputees; blind athletes)**		
Mean TLC L (SD)	6.54 (1.57)	5.34 (2.03)	7.40 (1.80)	6.73 (1.73)	0.11
Mean RV L (SD)	2.35(0.87)	2.03 (0.49)	2.57 (1.21)	2.40 (0.97)	0.64
Mean FRC L (SD)	3.34 (1.06)	2.57 (0.74)	3.97 (1.46)	3.49 (1.23)	0.12
Mean VC L (SD)	4.19 (0.99)	3.31 (1.74)	4.84 (0.77)	4.34 (1.04)	0.03
Mean ERV L (SD)	0.99 (0.44)	0.54 (0.38)	1.40 (0.48)	1.09 (0.50)	<0.01
Mean FVC L (SD)	4.01 (0.88)	3.14 (1.76)	4.74 (0.77)	4.18 (1.00)	0.01
Mean FEV1 L (SD)	3.48 (0.68)	2.80 (1.75)	4.10 (0.68)	3.63 (0.83)	0.01
Mean NO ppb (SD)	31.2 (26.4)	51.7 (30.8)	25.4 (7.8)	30.8 (22.9)	0.24

Legend: *L* = liters; *SD* = standard deviation; *TLC* = total lung capacity; *RV* = reserve volume; *FRC* = functional reserve volume; *VC* = vital capacity; *ERV* = endexpiratory reserve volume; *FVC* = forced vital capacity; *FEV1* = forced expiratory volume in one second; p-values are given for the difference between the three neurologically defined groups (one-way ANOVA).

### Exhaled NO

Exhaled NO did not differ significantly between the three neurologically defined groups (Table 2), nor between athletes with or without pre-existing asthma. There was also no difference in the exhaled NO between athletes with or without exercise-induced bronchoconstriction (either MCT or EVH challenge test positive) (25.2 ppb vs. 33.1 ppb, p = 0.53). However we found a statistically significant difference for athletes with atopy. These subjects had significantly higher values of exhaled NO than those without atopy (41.8 ppb, vs. 25.6 ppb, p = 0.03).

### EVH challenge

Forty-one athletes completed the EVH challenge with a mean ventilation of 82% MVV. Two athletes failed to reach the proposed 65% MVV for an adequate test, but ventilated not less than 63% of MVV. One athlete did not complete the test. Nine subjects (21%) had a positive EVH challenge. Of these, eight were paraplegics with a mean fall in FEV1 of 15.74% (SD 5.4%) and one was tetraplegic with an 11.6% fall in FEV1. None of the athletes in group 3 had a positive test. Those with a negative test had a mean fall in FEV1 of 4.3% (SD 2.6%).

Seven of the paraglegic athletes and the tetraplegic athlete had a diagnosis of asthma. A positive EVH challenge was significantly more common in athletes with asthma than without: eight athletes of eleven with a positive history of asthma had a positive EVH challenge compared to only one without a history of asthma (p < 0.001) (Table 3). The positive predictive value of EVH to identify physician diagnosed asthma was 89%, the negative predictive value 91%.

**Table 3:** Results of EVH and MCT

**Subject nr.**	**Diagnosis of asthma**	**Group**	**EVH: fall in FEV1 (%)**	**MCT: fall in FEV 1 (%)**
1	No	1	5.6	0.0
2	No	3	1.3	1.9
3	No	3	2.8	7.8
4	No	2	4.8	0.0
6	No	3	0.9	8.5
7	No	1	2.6	5.3
9	No	1	6.8	5.6
10	No	1	5.9	8.1
11	No	1	4.2	0.6
12	No	3	4.9	5.8
13	No	1	4.4	10.0
14	No	3	5.3	6.3
15	No	3	0.8	0.0
18	No	1	5.0	4.1
21	No	3	2.1	3.1
22	No	2	9.4	7.0
23	No	1	3.0	0.0
24	No	1	3.7	0.0
26	No	1	1.9	0.7
27	No	3	0.5	2.5
28	No	1	0	5.8
29	No	3	7.9	6.0
30	No	3	9.1	4.1
31	No	1	3.3	8.8
32	No	1	No test available	3.9
33	No	1	1.3	1.9
35	No	1	4.1	10.7
36	No	3	5.3	3.8
37	No	1	16.9	5.1
38	No	3	3.6	4.8
39	No	1	5.9	2.1
40	No	3	2.3	7.0
42	No	1	3.6	9.6
5	Yes	1	7.3	12.7
8	Yes	1	13.5	2.0
16	Yes	3	8.9	8.2
17	Yes	1	10	11.1
19	Yes	1	27.2	19.4
20	Yes	1	12.8	5.2
25	Yes	1	14.3	0.0
34	Yes	2	11.6	13.5
41	Yes	1	8.5	14.9
43	Yes	1	18.4	21.5
44	Yes	1	12.8	7.8

Group 1: paraplegic; group 2: tetraplegic; group 3: blind or single limb amputation.

### MCT challenge

All subjects completed the challenge. Eight athletes (18.2%) showed a fall in FEV1 > 10% after inhaling a maximal cumulative dose of 635 mg of Mannitol, with a mean fall of 13% (SD 4.0%) (Table 3). The geometric mean value for PD_10_ was 313 mg. Of the eight athletes with a positive MCT, seven were paraplegic and one tetraplegic. The mean maximum percentage fall in FEV1 in athletes that had a negative MCT was 4.53% (SD 3.8%).

Six of eleven athletes with a positive history of asthma had a positive test result in MCT compared to two athletes without asthma (p = 0.001). The positive and negative predictive values of MCT to identify physician diagnosed asthma were 75% and 86%, respectively.

### Comparison between MCT and EVH challenge

**Figure 2:**
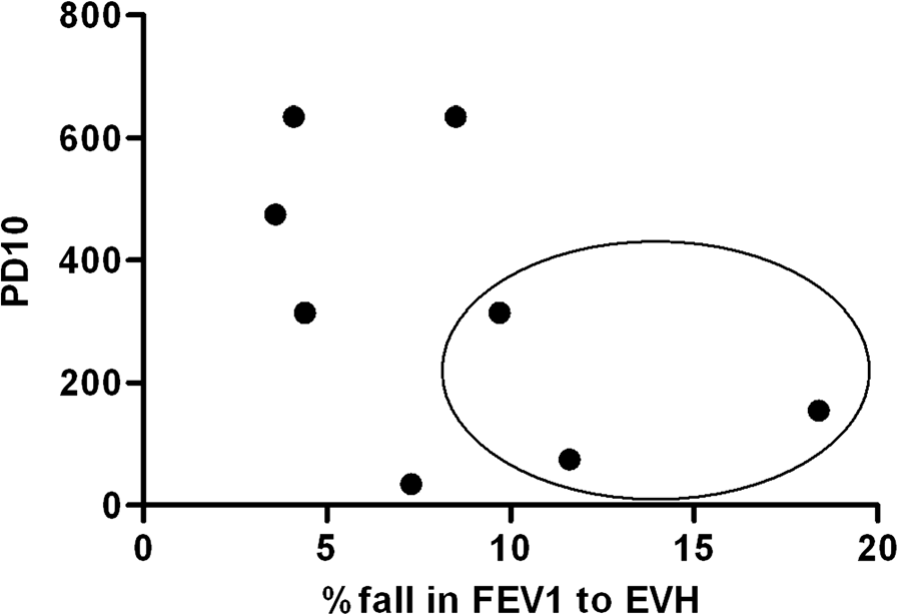
**Comparison of EVH challenge to mannitol challenge.** PD10 = provoking dose 10: cumulative dose in mg of Mannitol to cause a 10% fall in FEV1 (forced expiratory volume in one second); EVH = eucapnic voluntary hyperventilation; results of athletes with positive tests on both challenges are circled.

**Figure 3:**
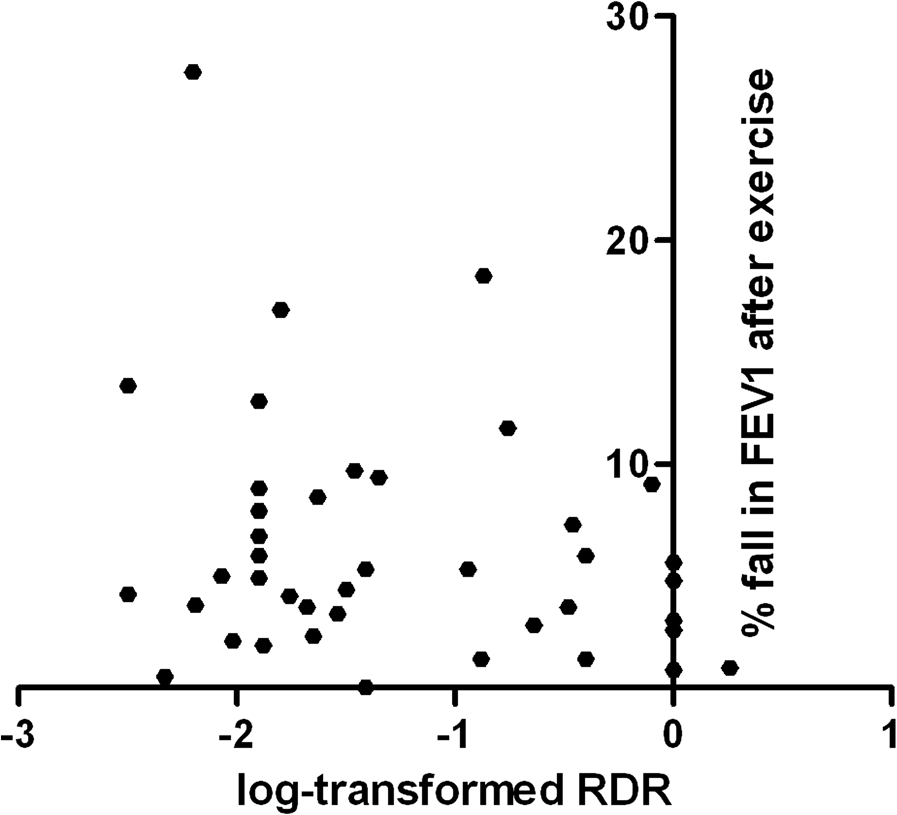
**Comparison of EVH challenge to mannitol challenge.** FEV1 = forced expiratory volume in one second; RDR = response dose ratio.

Nine participants had a positive response to EVH and eight had a positive response to the MCT. Ten of the eleven athletes diagnosed with asthma had at least one positive test for EVH or mannitol challenge. However, only three athletes had a positive response to both challenges resulting in a kappa of 0.19. Neither log-transformed RDR for mannitol and fall in FEV1 after exercise showed any correlation (r = -0.26; 95% CI: -0.53 to 0.043) nor log- transformed values of PD10 mannitol challenge and percentage of fall in FEV1 after EVH (r = -0.39; 95% CI: -0.86 to 0.44) (Figure 2 and 3).

## Discussion

This is the first study reporting the feasibility of bronchial challenge testing with EVH and MCT in athletes with disabilities investigating for the existence of exercise-induced bronchoconstriction or asthma. Ten of the eleven athletes diagnosed by a physician with asthma had at least one positive test for EVH or MCT challenge whereas three athletes with asthma had a positive response to both challenges. EVH and MCT had a high negative predictive value and thus a negative challenge test would allow to exclude asthma in this population.

Baseline lung function testing showed significant differences in VC, ERV, FVC and FEV1 between the groups with spinal cord injury and the group of athletes with other disabilities but no spinal cord injury. Athletes with tetraplegia had lower lung function values compared to athletes with paraplegia. Previous research reports that injury to the cervical and upper thoracic spinal cord decreases the function of inspiratory and expiratory respiratory muscles while the expiration is more impaired in individuals with tetraplegia or paraplegia and high spinal cord lesion [19-21].

The measured exhaled NO did not differ significantly between athletes with spinal cord injury and athletes without spinal cord injury, nor did it differ between subjects with or without physician diagnosed asthma. However the group of tetraplegic individuals tended to have higher NO levels compared to those with lesions leading to paraplegia. This is in concordance with another study reporting elevated levels of exhaled NO in tetraplegic individuals that were in the range of NO levels expected for individuals with mild asthma [22]. However it is currently unclear if elevated exhaled NO levels in tetraplegic individuals are a result of increased activity of the inducible NO synthetase due to inflammation or due to an altered regulation of inducible NO synthetase [22].

In the 2012 version of the world anti-doping agency prohibited list the use of all beta-2 agonists with the exceptions of salbutamol, formoterol and salmeterol are prohibited and athletes that use these substances must apply for exemption and provide objective criteria for the existence of exercise-induced bronchoconstriction or exercise induced asthma [23].

To our knowledge, this is the first study to evaluate exercise-induced bronchoconstriction by EVH or mannitol in athletes with spinal cord injury. As exercise challenge testing is especially difficult to standardize in this group of elite athletes, EVH and inhalation of dry powdered mannitol are two options to diagnose exercise-induced bronchoconstriction and allow the therapeutic use of inhaled beta agonists during competition. Both challenge tests showed a significant association with physician diagnosed asthma. Both EVH and MCT were shown to have a high negative predictive value which suggests that a negative test can reliably exclude asthma in this population of athletes with disabilities. The diagnostic validity of MCT in our study is similar to those previously reported in able-bodied athletes [10,24,25].

In our group, 21% of all participants were identified as having exercise-induced bronchoconstriction by eucapnic hyperventilation. In other studies testing able-bodied elite athletes exercise-induced bronchoconstriction was identified in 31% to 50% [10,26-28] by EVH challenge. This difference cannot be explained by inadequate hyperventilation as the minimum achieved ventilation equalled 63% maximum voluntary volume and the mean ventilation was 82% MVV. Only two athletes failed to reach the proposed 65% MVV for an adequate test. The lower proportion of positive EVH tests is in concordance with a relatively low prevalence of physician diagnosed asthma in our study sample. With the mannitol challenge, 18% tested positive for exercise-induced bronchoconstriction, compared with 52% in an Australian able-bodied elite athlete population [10]. None of the athletes without spinal cord injury had a positive test result in our study group. The difference might be explained by the inclusion of athletes of various sporting disciplines, as substantial differences in the prevalence of exercise-induced asthma have been reported, depending on the type of sport performed [29,30]. Low prevalences of hyperresponsiveness to mannitol were previously reported for a population of elite cross country skiers [17] as well as Scottish elite swimmers [24].

In the past, it was shown that the majority of patients with tetraplegia demonstrate airway hyperresponsiveness to methacholine [31,32], histamine [33] and ultrasonically nebulized distilled water [34]. One of the three tetraplegic athletes tested positive on MCT as well as on EVH. This athlete showed the lowest vital capacity and FEV1 of all athletes tested. It has been postulated that with small airway diameters a small further reduction in calibre induced by a bronchoconstrictive agent produces a large increase in resistance as airway resistance is inversely proportional to the fourth power of the radius [35]. The reduced airway calibre in these athletes does probably explain why a larger proportion of athletes with tetraplegia demonstrate airway hyperresponsiveness in the MCT and EVH.

When testing 50 elite able-bodied athletes, Holzer et al. demonstrated a strong association between the response to MCT and to the EVH [10]. In our study, we identified eight and nine athletes with a positive test result, respectively. However, only three athletes had congruent results on both challenges. Holzer et al. set the cut-off defining a positive test at >10% fall in FEV1 in the MCT [10]. In a study testing children for bronchial hyperresponsiveness, Kersten set the cut-off for a positive test result at > 15% in FEV1 in the MCT and concluded that the MCT was especially useful to exclude exercise-induced bronchoconstriction [36]. For children, different cut-off points for exercise testing and inhalational provocation tests are more suitable [37-39]. Had we defined a 15% cut-off in our subjects, only 2 participants would have tested positive on the MCT with a single athlete testing congruently positive on the MCT and the EVH. With only adults included in the study, we chose a cut-off of ≥10% fall in FEV1 in both challenge tests.

A significant relationship between the reactivity to mannitol, defined by the response–dose-ratio (RDR), and the fall in FEV1 after EVH could not be demonstrated in our study. While Kersten and co-workers [36] showed a strong relationship between RDR mannitol and the fall in FEV1 after exercise in able-bodied children, Clearie and co-workers could not find an association between reacitivity to mannitol and standardized field-based testing in elite swimmers [24].

## Conclusion

In conclusion, we present results of bronchial hyperresponsiveness testing in a population of elite athletes with a disability. EVH and MCT were found to be feasible and safe in diagnosing exercise-induced asthma in athletes with spinal cord injury. A combination of both challenge tests is recommended in this group of subjects. Eucapnic voluntary hyperventilation and inhalation of dry powder mannitol can be used to identify, but especially exclude asthma in Paralympic athletes due to their relative high negative predictive value for the diagnosis of physician-diagnosed asthma.

## Competing interests

The authors declare that they have no competing interests.

## Authors’ contributions

FM, MS, JDL and CP contributed substantially to conception and design, FM, MS and CP to acquisition of data, MO, FM, MS, DM, ABT and JDL to analysis and interpretation of data; MO, DM and ABT to drafting the article and all authors to revising it critically for intellectual content.

## Pre-publication history

The pre-publication history for this paper can be accessed here:

http://www.biomedcentral.com/2052-1847/5/7/prepub
